# Data-Driven Modeling of Friction in Drawbead Test Through Advanced Machine Learning

**DOI:** 10.3390/ma19122641

**Published:** 2026-06-18

**Authors:** Tomasz Trzepieciński, Romuald Fejkiel, Marek Kowalik

**Affiliations:** 1 Faculty of Mechanical Engineering and Aeronautics, Rzeszów University of Technology, al. Powstańców Warszawy 8, 35-029 Rzeszów, Poland; 2 Department of Mechanics and Machine Building, The University College of Applied Sciences in Krosno, ul. Wyspiańskiego 20, 38-400 Krosno, Poland; romuald.fejkiel@pans.krosno.pl; 3 Faculty of Mechanical Engineering, Casimir Pulaski Radom University, 54 Stasieckiego Street, 26-600 Radom, Poland; m.kowalik@uthrad.pl

**Keywords:** coefficient of friction, draw bead, draw bead simulator, friction, sheet metal forming, steel sheets

## Abstract

Friction at the drawbead in metal forming operations directly affects the quality of drawpieces. However, identifying the complex effect of friction process parameters on the coefficient of friction (CoF) is difficult based on experimental results. The aim of this paper is to analyze the results of a drawbead simulator test using various machine learning (ML) methods to select the most appropriate algorithm and to analyze in detail the feature importance, permutation importance, and cumulative Shapley additive explanation values of predictors. The test material was DC04 low-carbon steel sheet. Experimental tests were conducted for varying friction process conditions. Of the three different ML algorithms (support vector machine, regression trees, and ensemble tress), the support vector machine (SVM) algorithm with a cubic kernel function provided the lowest root mean square error (0.0085) and the highest correlation coefficient R^2^ (0.9657) for the test data. The predictors in descending order of permutation importance are friction conditions, drawbead height, sample width, Sa of countersamples, and sample orientation. A combined swarm-box chart presenting Shapley values for an SVM model with a cubic kernel function indicates that a low value of the drawbead height predictor has a strong, increasing effect on CoF. However, low values of the remaining explanatory parameters (sample width, mean roughness of countersamples, and sample orientation) have a decreasing effect on CoF.

## 1. Introduction

Friction is a fundamental phenomenon in metal forming processes, governing the interaction between the blank and the tooling surfaces. During deep drawing, the sheet metal slides against the stamping tool, generating frictional forces at the contact interfaces [1]. Friction primarily causes deterioration of the surface quality of the drawpieces [2]. The coefficient of friction (CoF) depends on many parameters, such as sheet metal and tool topography [3,4], lubrication conditions [5,6], contact pressure [7,8], temperature [9], and properties of the sheet material [10]. Therefore, understanding and accurately modeling friction is critical for optimizing sheet metal forming process parameters [11]. Lubrication in sheet metal forming processes plays a key role in controlling friction conditions at the tool–material interface, directly influencing the distribution of plastic strain and process stability [12,13]. A properly selected lubricant reduces tool wear, minimizes the risk of adhesion phenomena, and increases the efficiency of sheet metal forming operations [14]. Lubrication strategies are often optimized to achieve a controlled and consistent friction level [15].

In sheet metal forming, drawbeads are widely used to control material flow and improve part quality during deep drawing operations. A drawbead is a geometric feature located along the perimeter of the workpiece [16]. Its purpose is to control material flow in areas of workpiece prone to wrinkling by introducing tensile stresses in the sheet material. Material flow is modified by using a drawbead of appropriate height and geometry [17]. The total restraining force in a drawbead is generally composed of bending–unbending resistance and frictional resistance. Too low a drawbead height may result in insufficient restraint, while too high a drawbead resistance can lead to the risk of tearing [18].

Understanding friction in drawbeads is therefore essential for accurate process design. A drawbead simulator test is commonly used to evaluate friction behavior under realistic forming conditions [19,20]. Theoretical foundations for controlling the flow of sheet metal into the die cavity during sheet metal forming were presented by Nine [19] and Demeri [21]. Strain hardening occurs when the sheet passes through a drawbead, which changes the plastic properties of the sheet material [22]. Repeated bending and straightening of the sheet during the drawbead test also changes the sheet topography. Flattening of the surface asperities due to contact pressure is found to be the main influencing factor on the CoF [23]. The influence of the geometry and shape of the drawbead on material flow through a drawbead was determined by Triantafyllidis et al. [24]. They found that deep, narrow beads provide the greatest restraining force during the pulling phase of deformation. Meanwhile, wide, shallow beads allow material to pull through more easily as the drawpiece is formed. When examining sheet metals, the directionality of the rolling-induced microstructure of sheets must be taken into account. Coefficient of friction for strip samples cut in the sheet rolling direction were lower than for samples cut across the rolling direction [25]. Hance and Walters [26] found that sheet thickness significantly affects the CoF determined by the drawbead simulator test. Therefore, this test should be used to compare the frictional properties of materials of similar mechanical properties and thickness.

Although experimental studies provide the most reliable results, numerical finite element (FE) method simulations are commonly used to analyze sheet metal deformation in the drawbead region. Chabrand et al. [27] presented one of the first studies on numerical determination of the drawbead forces. These authors reported that the contact pressure distribution is highly non-homogeneous on the drawbead. Hussein et al. [28] numerically investigated the effect of using a drawbead on the thickness distribution along the cup. Comparing the results of FE-based simulations with the experimental results, the average error was 9.9%. Data processing and prediction algorithms were used to analyze experimental data and the results of numerical simulations of friction phenomena [29].

In addition to experimental methods, artificial intelligence (AI) techniques are increasingly being used to analyze complex friction phenomena. Machine learning (ML) is an AI technique that allows for the discovery of relationships in training data [30]. With the rapid increase in data generation, ML tools are crucial in tribology, allowing for the identification of trends and the determination of complex interactions between parameters affecting the coefficient of friction [29]. Machine learning algorithms can be divided into three basic types: supervised learning, unsupervised learning, and reinforcement learning [31,32]. The most common type of ML is supervised learning, which includes linear regression, decision trees, random decision forests, support vector machines, k-nearest neighbors, and others. In the field of tribology, ML algorithms have been successfully used, among others, for the analysis of friction in sheet metal forming [29], performance optimization of novel tribological materials [33], selection of lubricant formulations [34], lubrication regime classification [35], predicting lubricated friction on textured surfaces [36], prediction of nanoscale friction for two-dimensional materials [37], and wear performance [38].

Overall, friction in the drawbead region is a key parameter in controlling metal flow, improving part quality, and preventing common forming defects. A balanced understanding of frictional mechanisms enables engineers to design more robust and efficient sheet metal forming processes. Compared to friction testing using strip drawing tests, drawbead simulators are used in a niche setting; therefore, identifying the friction behavior of sheets in the drawbead zone still requires in-depth analysis. The results of friction tests of sheets using the drawbead simulator test are very scarce in the literature and only a few works address the topic of using ML to model the relationships between inputs and COF determined using the drawbead simulator. To the best of the authors’ knowledge, this study is the first to perform both support vector machine-based feature importance analysis and permutation importance analysis using data obtained from drawbead friction tests. A comprehensive comparison of the predictive capabilities of three major machine learning algorithms: support vector machines, regression trees, and ensemble trees, has been also conducted. This approach allowed us to identify the limitations of various machine learning approaches to modeling the friction phenomenon at the drawbead in sheet metal forming. The complex interaction between drawbead geometry and lubrication regime makes friction behavior nonlinear and challenging to model precisely [39]. Therefore, this paper proposes the use of machine learning (ML) methods for predictive and sensitive data-driven modeling of friction in drawbead. Machine learning algorithms and Shapley Additive exPlanations (SHAP) analysis can identify relevant forming parameters and supports decision-making processes based on empirical data [40,41,42]. Experimental data from a drawbead test conducted on low-carbon DC04 steel sheets, commonly used in the automotive industry, were used as the input data. In our previous work [29], the random forest ML algorithm was used to analyze the friction of EN AW-5251 aluminum alloy sheets. The inputs analyzed were the yield strength of sheet metal R_p0.2_, average roughness of countersamples Ra, oil viscosity, and strength coefficient of sheet material K. CoF was considered as the output. Due to the difference in input parameters, it is difficult to compare the approach presented in this work and in [29]. However, in [29], models characterized by the coefficient of determination *R*^2^ between 0.46 and 0.86 were obtained, depending on the neuron activation function.

## 2. Material and Methods

### 2.1. Material

**Figure 1:**
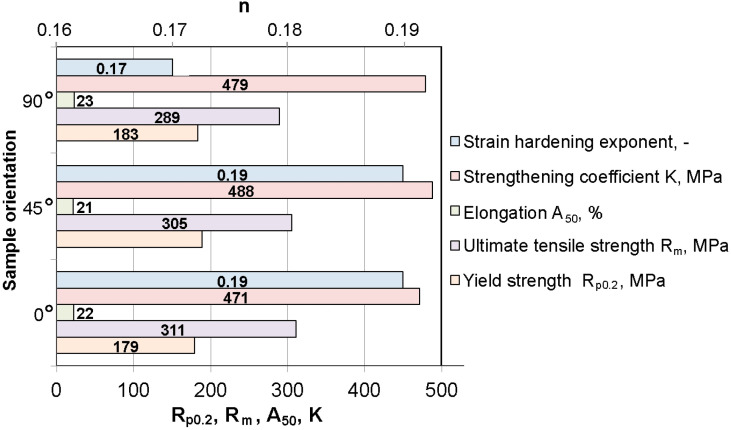
Basic mechanical parameters of DC04 steel sheet.

Experimental tests were conducted on samples of DC04 low-carbon steel, classified according to the EN 10130 standard [43]. The sheet thickness was *t* = 0.8 mm. DC04 steel is characterized by a very low carbon content (typically ≤ 0.08 wt.%). The main criterion for selecting this material was that it is commonly used in industrial processes of forming geometrically complex drawpieces requiring the use of drawbeads. The basic mechanical parameters of the DC04 steel sheet were determined by uniaxial tensile testing of samples cut in three directions, that is, in the rolling direction (RD), transversely to the RD, and at an angle of 45° to the RD. Tests were conducted in accordance with the requirements of the ISO 6892-1:2020 standard [44]. The mechanical properties of this steel include a relatively low yield strength, moderate tensile strength, high elongation, as well as favorable strain hardening parameters (Figure 1), determining its susceptibility to deep drawing operations. Average values of basic mechanical properties were determined based on five replicates.

**Figure 2:**
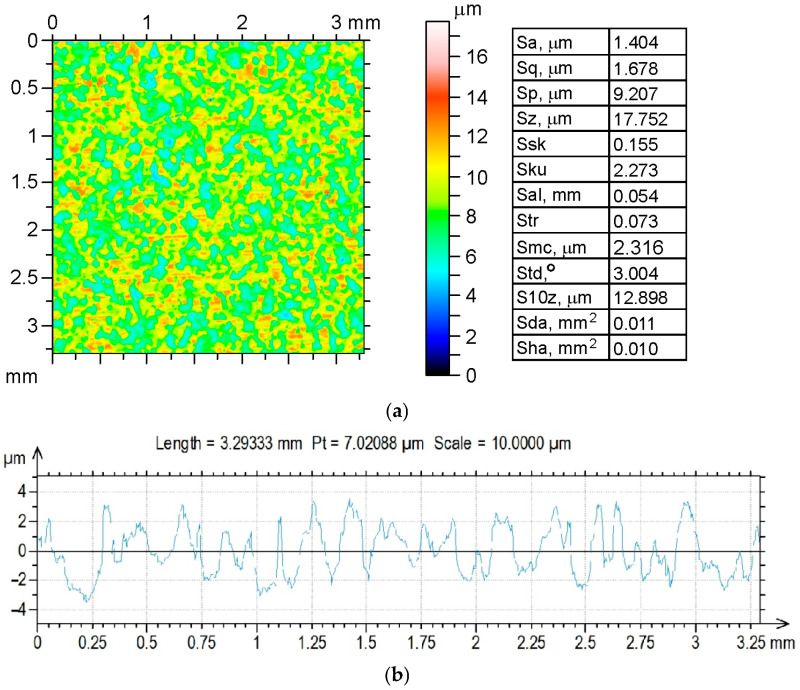
(**a**) surface topography, basic surface roughness parameters, and (**b**) surface roughness profile of DC04 steel sheet.

The topographic map (Figure 2a), surface roughness profile (Figure 2b), and basic surface roughness parameters were determined using a Talysurf CCI Lite interferometer (Taylor Hobson Ltd., Leicester, UK) with a resolution of 0.01 nm. Roughness parameters were determined according to the ISO 25178-6 standard [45].

### 2.2. Experimental Procedure

**Figure 3:**
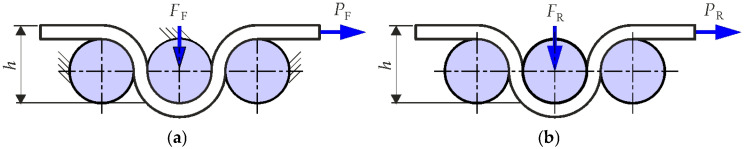
Schematic diagram of drawbead simulator test with (**a**) fixed and (**b**) freely rotating countersamples.

Friction tests to determine the CoF at the drawbead region in sheet forming operations were carried out using a drawbead simulator, which was developed by the authors based on the theoretical concept proposed by Nine [19]. The proposed concept involves drawing a sheet metal strip through a system of fixed rollers (Figure 3a) and a system of freely rotating rollers (Figure 3b). This strategy allows for separating frictional resistance from resistance associated with plastic deformation of the samples. During both stages, the normal forces *F*_F_, *F*_R_ and pulling forces *P*_F_, *P*_R_ were recorded.

**Figure 4:**
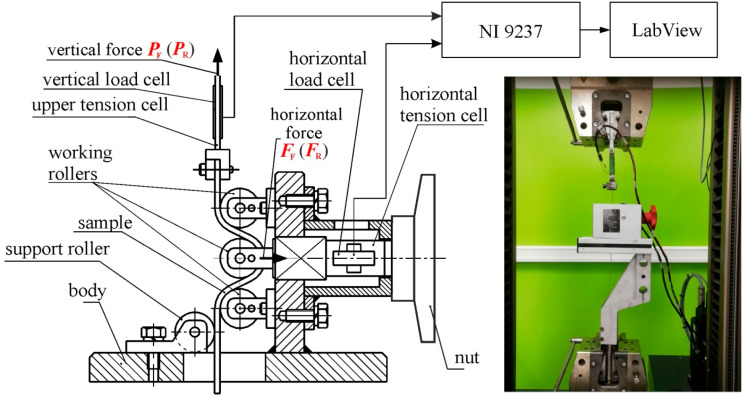
Diagram and view of the testing device (process forces are marked in red).

The drawbead simulator (Figure 4) consists of a body in the bottom grip of the universal Zwick/Roell Z100 tensile testing machine (Zwick/Roell GmbH & Co. KG, Ulm, Germany). Three rollers with a radius of 10 mm replicate the drawbead. This design allowed the rollers to be fixed from freely rotating. The support roller prevented bending of the free end of the strip sample. The rollers were made of 145Cr6 tool steel. The appropriate drawbead height was adjusted using nut. The tribotester body was mounted in the bottom grip of a Zwick/Roell Z100 tensile testing machine. While the sheet metal strip was being pulled through the system of rollers at a speed of 10 mm/s, the process forces were measured using tension cells. The signals from the tension cells were recorded using the NI 9237 Bridge Input Module with a frequency of 50 Hz. The strip samples were 400 mm long. The width of the specimens determines the character of the sheet strip deformation during passage through the system of rollers [46]. Therefore, sheets of various widths were tested, i.e., 6, 12, and 18 mm. Additionally, to assess the effect of sample orientation on the CoF, strip samples cut along the RD and transversely to the RD were tested.

A discussion of the technical details of the tribotester design and research methodology can be found in the authors’ previous works [46]. This article focuses on the analysis of drawbead test results using machine learning methods.

The CoF was determined from the formulae [47]:

(1) $$\mu =\frac{sin\alpha }{2\alpha }\times \frac{P_{F}-P_{R}}{F_{F}}$$

**Figure 5:**
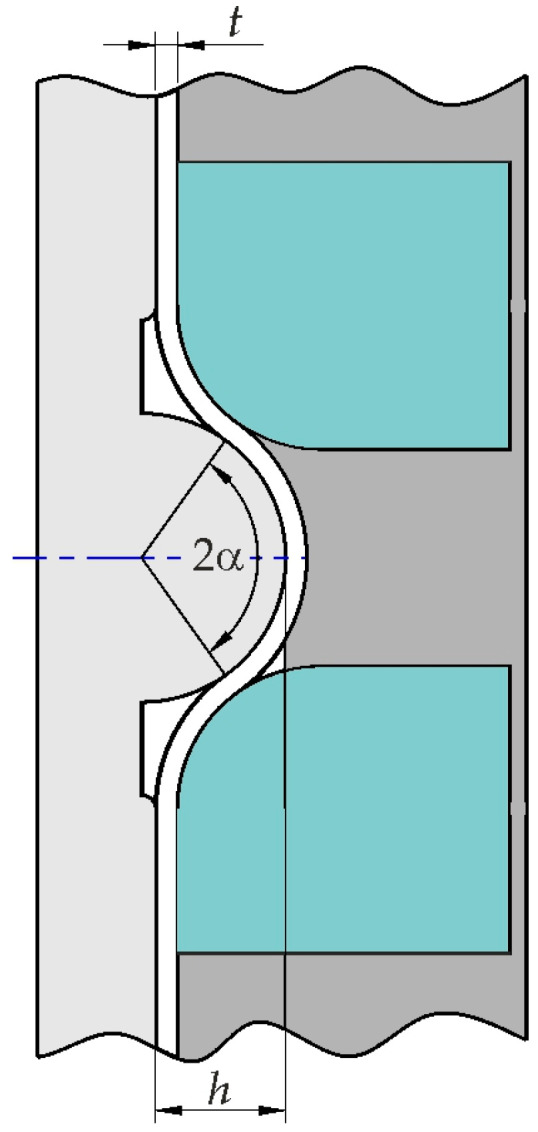
Geometric parameters of the drawbead.

*F*_F_ is the horizontal force obtained with fixed rollers, *P*_R_ is vertical force obtained with the freely rotating rollers, *P*_F_ is vertical force obtained with fixed rollers, and 2α is the contact angle (Figure 5) equal to 36.4°, 82.6°, and 111.4° for drawbead heights *h* of 6 mm, 12 mm, and 18 mm, respectively.

**Figure 6:**
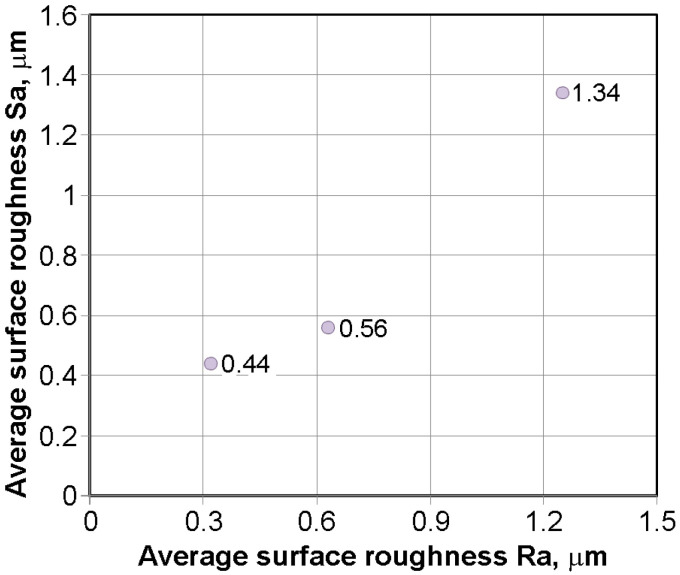
Relationship between Ra and Sa parameters of countersample surfaces.

Three sets of countersamples with the following average surface roughness Sa were used in the tests: 0.44 μm, 0.56 μm, and 1.34 μm. The countersample surfaces were machined to provide typical standardized average roughness values of Ra: 0.32 μm, 0.63 μm, and 1.25 μm (Figure 6). During machining, this parameter was measured along the roller surfaces, facilitating the selection of appropriate machining parameters to achieve the desired roughness. However, in tribological applications involving friction, the average surface roughness (Sa) is more relevant in the analysis of contact phenomena [48]. Therefore, the average roughness Sa was considered in the analysis of the effect of friction parameters on CoF.

Tests were conducted under the following conditions (samples and rollers were cleaned with acetone prior to testing):

- dry friction,
- LAN-46 machine oil with a kinematic viscosity of η = 43.9 mm^2^/s,
- Heavy-Draw (HD) 1150 metal forming lubricant (η = 1157 mm^2^/s).

### 2.3. Machine Learning

Regression in machine learning is a method for predicting the numerical values of a dependent variable, typically based on multiple explanatory variables. Regression allows to determine how independent variables influence the dependent variable, allowing for the identification of trends and relationships between variables. Linear regression is also a method of supervised ML. Statistical modeling and ML are two commonly used analytical approaches for identifying patterns in data. Statistical modeling is based on building interpretable models that explain the relationships between variables, assuming a specific data structure. ML methods, on the other hand, focus on maximizing predictive accuracy using algorithms, often without the need to understand the internal structure of the data.

**Figure 7:**
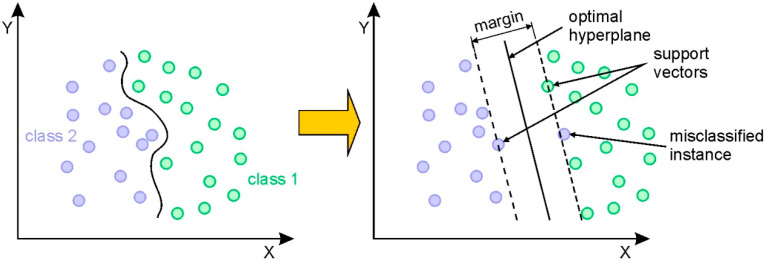
Idea of SVM optimal hyperplane.

Two main ML algorithms were used to analyze experimental data for determining the CoF of the DC04 sheet metal: support vector machine (SVM) and ensembles of trees. The SVM performs classification tasks by constructing hyperplanes in a multidimensional space that separate instances belonging to different classes [49]. However, regression can also be performed by SVM, and both of these tasks can be performed for multiple variables, both numerical and categorical. SVM is based on the concept of a decision space, which is divided by constructing boundaries separating objects with different class affiliations (Figure 7). SVM performs classification tasks by constructing hyperplanes in a multidimensional space that separate instances belonging to different classes [50]. This hyperplane is defined to maximize the distance between the closest examples of both classes and the hyperplane itself. The SVM algorithm demonstrates the ability to generalize in the face of the challenges associated with high-dimensional data. In regression applications, the goal of SVM is to find the function that best describes the relationship between the features and the target variable while minimizing prediction errors.

Regression trees are a type of decision tree. They use sum-of-squares and regression analysis to predict the value of a dependent variable [51]. Predictions are based on a combination of input variable values. Each node of the tree is split into two or more child nodes to reduce the sum of squares for that node. Child nodes corresponding to specific predictor categories are merged, and for each node, the predictor that reduces the sum of squares the most is selected for splitting. Each node of the tree is split into child nodes until the sum of squares can no longer be improved [52]. A regression tree strives to provide a set of rules with the smallest prediction error.

**Figure 8:**
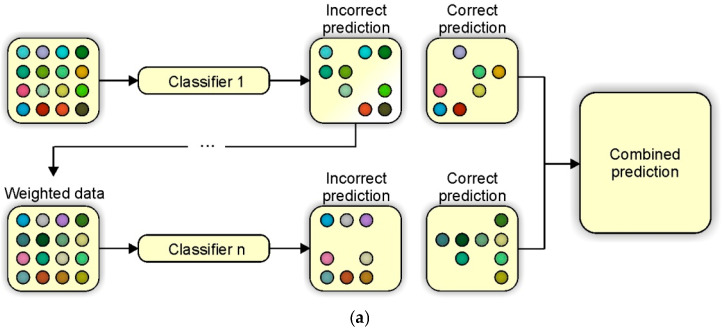

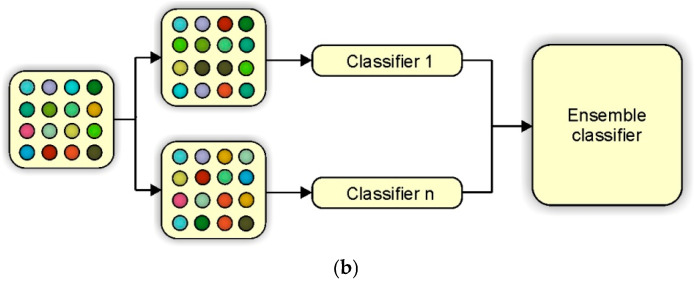
Visual explanation of (a) boosting and (b) bagging.

Ensemble tree is a supervised machine learning technique that individually trains decision trees [53,54]. This involves aggregating multiple models with weak performance into a new, strong model. Ensemble methods have a greater ability to generalize and reduce errors resulting from underfitting or overfitting, making them particularly useful for unstable datasets. A key advantage of this approach is the ability to reduce errors resulting from overfitting or underfitting that can occur with individual models. This paper utilizes two distinct ensemble learning methods: boosting and bagging. Boosting is based on an iterative approach in which trees are trained sequentially [55] (Figure 8a). Misclassified observations in previous learners are assigned a higher weight. In this way, each successive tree is built sequentially [56]. Boosting focuses on correcting the errors of the predecessors, which results in a reduction of the overall model error. In the bagging (Bootstrap AGGregation) ML method, each decision tree uses a bootstrap sample of the training data set as input data [57]. A bootstrap sample is a randomly selected sample with replacement. By combining multiple independently trained trees (Figure 8b), bagging reduces model variance, limiting the risk of overfitting. By averaging the results of multiple models, bagging helps increase stability and prediction accuracy.

ML algorithms were used to analyze the relationships between friction process parameters and the CoF. The explanatory variables included:

- friction condition (categorical input—Table 1),
- sample orientation relative to the RD (categorical input—Table 1),
- Sa of countersamples (numerical input),
- drawbead height (numerical input),
- width of the sample was considered (numerical input).

Various types of SVM, regression tree, and ensemble tree algorithms listed in Table 2, Table 3 and Table 4 were considered.

From the experimental dataset (162 sets), a test set containing 10% of the data was selected to evaluate the predictive capabilities of the ML algorithm. The remaining dataset was used in the training process. To prevent overfitting, 10-fold cross-validation was applied.

## 3. Results and Discussion

### 3.1. Regression Statistics of SVM Models

Root Mean Square Error (RMSE) is a fundamental scale-dependent metric for evaluating ML models in regression applications. RMSE assesses predictive accuracy by calculating the square root of the mean differences between predicted and observed outcomes [58]:

(2) $$RMSE=\sqrt{\frac{1}{n}\sum_{i=1}^{n}{\left(y_{p}-y_{t}\right)}^{2}}$$

where *y*_p_ is the predicted value, *y*_t_ is target value and *n* is the number of data points.

Scale-dependent metrics are favored in ML evaluation [59]. To obtain a comprehensive assessment of model quality, it is recommended to use RMSE in combination with the coefficient of determination *R*^2^ (Equation (3)) [58]. Such an approach provides a more confident understanding of model reliability. R^2^ interprets the degree of correlation between the predicted and actual variables. A range from 0.8 to 1 indicates a very strong correlation [59].

(3) $$R^{2}=\frac{\sum_{i=1}^{n}{\left(y_{t}-\bar{y}\right)}^{2}-\sum_{i=1}^{n}{\left(y_{p}-y_{t}\right)}^{2}}{\sum_{i=1}^{n}{\left(y_{t}-\bar{y}\right)}^{2}}$$

where $\bar{y}$ is the average value of *y*_t_.

**Figure 9:**
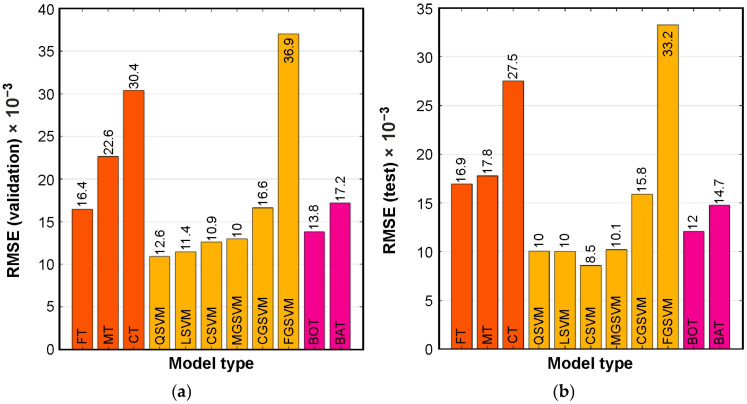
Effect of the ML model on the RMSE for the (**a**) validation and (**b**) test data (different groups of algorithms are marked with colors: regression trees—dark orange, SVM-based algorithms—yellow, boosted trees—pink).

The RMSE evaluated for the validation data (Figure 9a) can be used to assess the predictive quality of the training data. However, the RMSE determined for the test data (Figure 9b) is directly related to the prediction based on the predictor values that were not involved in the training process.

The RMSE errors for regression trees (CT, MT, FT) and ensemble trees (BAT, BO) are similar for each of these ML algorithms. Increasing the number of leaves in regression tree-based algorithms increases the RMSE (Figure 9a,b). A larger number of leaves provides a more detailed partitioning of the data but simultaneously limits the model’s generalization capabilities. Similarly, for the FGSVM algorithm with a small scale of 0.56, the RMSE is almost three times higher than for the other SVM algorithms (Figure 9a,b).

**Figure 10:**
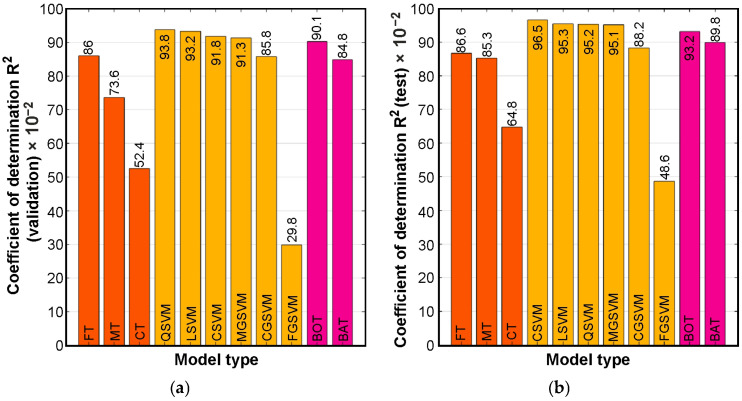
Effect of the ML model on the R-squared coefficient of determination for the (**a**) validation and (**b**) test data (different groups of algorithms are marked with colors: regression trees—dark orange, SVM-based algorithms—yellow, boosted trees—pink).

There is a clear relationship between the RMSE value (Figure 9) and the coefficient of determination (Figure 10): the smaller the RMSE, the higher the *R*^2^ value. Most of the analyzed ML models were characterized by a very strong correlation. Only in the case of three models (FGSVM, CT, and MT) the coefficient of determination was less than 0.8. The best predictive ability was demonstrated by SVM algorithms with the following kernel functions: linear, quadratic, and cubic. For these models, *R*^2^-values were greater than 0.918 and 0.952 for the validation (Figure 10a) and test (Figure 10b) data, respectively.

The CSVM model had the lowest RMSE value for the test set (RMSE = 0.0085). At the same time, this model was characterized by the highest correlation (*R*^2^ = 0.9657) for the data included in the test set (Figure 10b). Therefore, the CSVM model was selected for further detailed feature importance analysis.

Additionally, Table 5 presents a comparison of RMSE errors with other qualitative metrics of the analyzed ML models, including Mean Square Error (MSE) (Equation (4), Mean Absolute Error (MAE) (Equation (5), and Mean Absolute Percentage Error (MAPE) (Equation (6) [58,59]. MSE and MAE are scale-dependent metrics and MAPE is percentage-dependent metric [59].

(4) $$MSE=\frac{1}{n}\sum_{i=1}^{n}{\left(y_{p}-y_{t}\right)}^{2}$$

(5) $$MAE=\frac{1}{n}\sum_{i=1}^{n}\left(\left|y_{p}-y_{t}\right|\right)$$

(6) $$MAPE=\frac{1}{n}\sum_{i=1}^{n}\left|\frac{D_{pre}-D_{act}}{D_{act}}\right|\times 100\%$$

where *D*_pre_ is the predicted variable and *D*_act_ is the actual variable.

### 3.2. Predictive Performance of Cubic SVM Model

**Figure 11:**
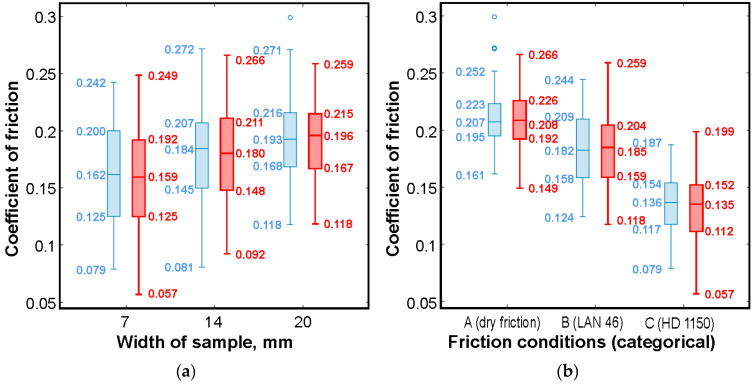

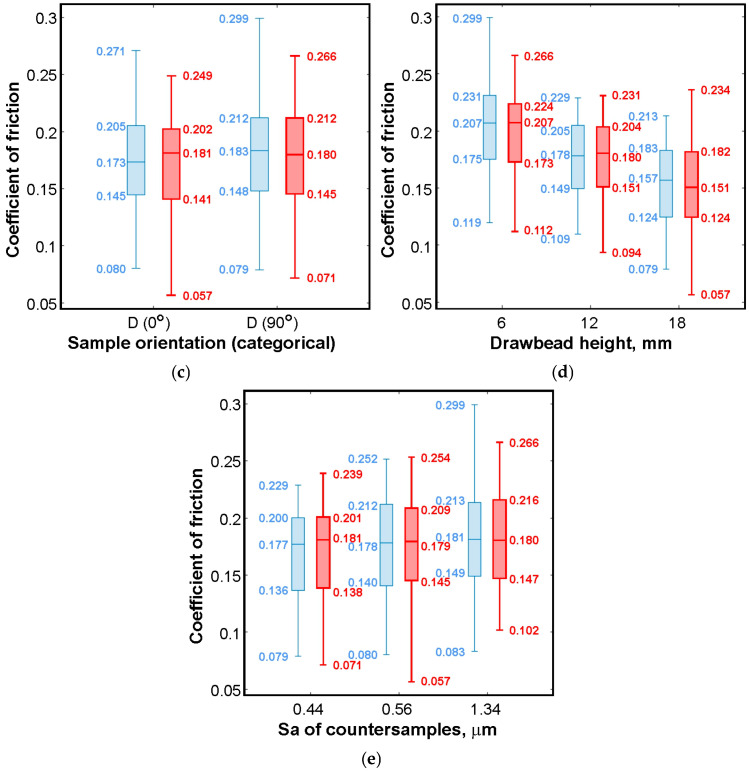
Response box plots for predictions of CoF for CSVM model): (**a**) width of sample, (**b**) friction conditions, (**c**) sample orientation, (**d**) drawbead height, and (**e**) Sa of countersamples (blue and red box plots correspond to true and predicted data, respectively).

The box plots in Figure 11 compare the experimental data with the CoF predictions using the CSVM model. The experimental data for all analyzed input parameters (Figure 11a–e) generally exhibit symmetry, manifested by the median being located near half the box height. Increasing the sample width increases the CoF value (Figure 11a). Considering the median, this increase is 87% for the analyzed range of sample width changes (from 0.162 to 0.193). Furthermore, the interquartile range between the first and third quartiles (50% of the data) decreases with increasing sample width (Figure 11a). This indicates less scatter in the data. The effect of sample width on the CoF in the drawbead test is related to the character of the material deformation during sequential bending and straightening of the strip sample in the drawbead test. This effect was numerically investigated in the authors’ earlier work [50]. Changing the strip sample width determines a different change in the real contact area between the sheet and the countersamples. The prediction of the CoF expressed by the interquartile range (box height) differs from the experimental values between 0.000 and 0.008 (Figure 11a).

The CoF prediction related to friction conditions (categorical variable) is characterized by slightly larger whiskers (Figure 11b). The ends of the lower and upper whiskers define the lowest and highest values in the data set, excluding outliers. Outliers are observations that are more than 1.5 interquartile ranges below the first quartile or above the third quartile [60]. HD 1150 oil demonstrated greater effectiveness in reducing the coefficient of friction compared to LAN 46 machine oil (Figure 11b). Considering the median values, HD 1150 oil provided a 34.3% CoF reduction compared to dry friction conditions. The use of lubricant was the most effective way to reduce the coefficient of friction [61]. The higher efficiency of HD 1150 oil compared to LAN 46 can be explained by the higher viscosity, which provides a thicker lubricating film, limiting the metallic contact of the summits of asperities.

Of all the input parameters analyzed, changing the sample orientation with respect to RD exhibited the least impact on CoF change. The difference in the median of the experimental results for both orientations is 0.01 (Figure 11c). Furthermore, the limiting values included in the boxplot heights do not exceed 0.007. The predictions for the data within the interquartile range between the first and third quartiles are in good agreement with the experimental data. The difference between the lower and upper values of the range is between 0 and 0.004.

Increasing the drawbead height causes a decrease in the average CoF value (Figure 11d). Increasing the degree of sheet deformation during sequential bending and straightening of the strip sample increases the sheet strength due to the work hardening phenomenon. Higher material strength limits the plastic deformation of the asperity summits and consequently reduces the flattening phenomenon [62]. The predictions are consistent with the box height and median position (Figure 11d).

The experimental results also showed that CoF increases with increasing average roughness of the countersamples (Figure 11e). The influence of countersamples roughness on CoF is multifaceted. On the one hand, lower countersamples roughness limits the phenomenon of mechanical scratching of the sheet metal surface, leading to a smaller intensity of metallic contact [63]. However, on the other hand, lower surface roughness is characterized by smaller spaces (valleys) that can act as a lubricant reservoir [64]. Therefore, the relationship between countersamples roughness and CoF comes down to assessing this effect on lubrication efficiency and mechanical interactions between the asperities of the friction pair.

**Figure 12:**
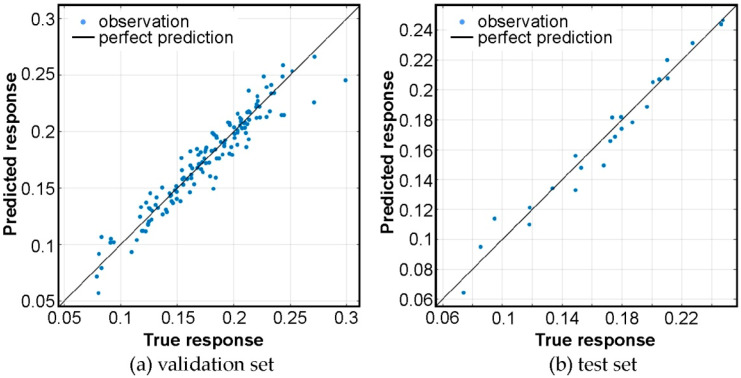
Comparison of actual vs. predicted CoF (CSVM model).

A comparison of the actual and predicted CoF values for the validation data and test data is shown in Figure 12a and Figure 12b, respectively. Data points are distributed evenly along and proportionally around the perfect prediction line. The determined R^2^-values for the validation and test data were 0.9183 and 0.9657, respectively. This indicates that the CSVM model can account for around 91.83% and 96.57% of the variation in the target variable within the validation and test data, respectively. This confirms that the CSVM model and test set are very well fitted.

### 3.3. Feature Importance Analysis

Feature importance analysis is a method for estimating the contribution of each feature to an ML model’s predictions. Feature selection is the process of evaluating the features in the model that have the greatest impact on the target variable. Permutation importance (PI) is a method used to determine the importance of features based on their impact on the predictions of a trained ML model. PI focuses solely on its predictive performance, ignoring the model’s internal behavior. Permutation importance assesses how information changes when the information carried by a feature is systematically perturbed. The resulting deterioration in predictive performance provides a measure of the feature’s importance to the ML model.

**Figure 13:**
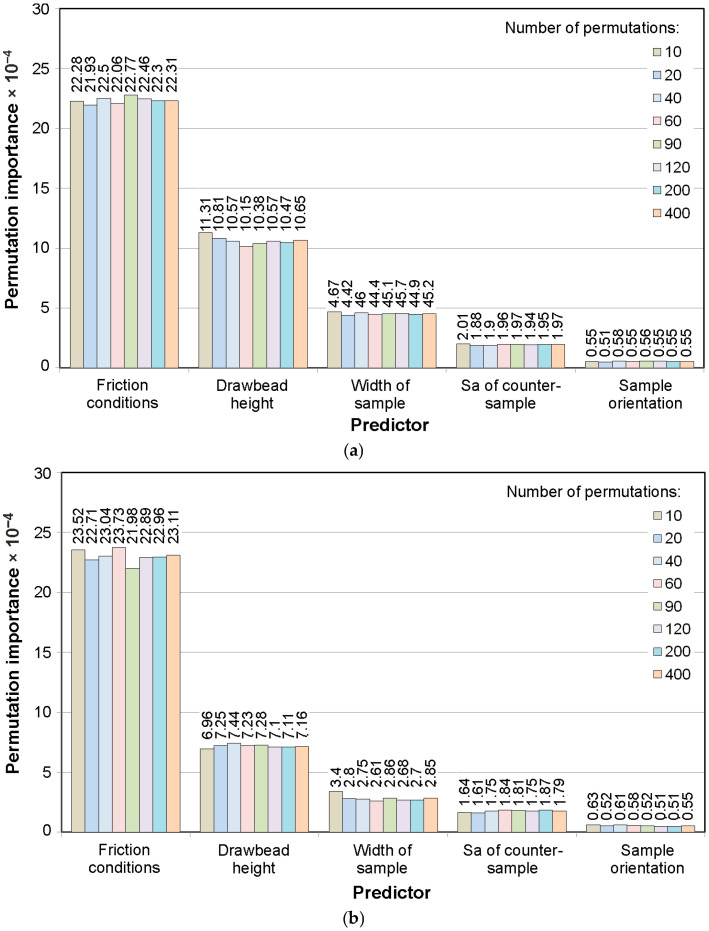
Permutation importance of CSVM model based on the (**a**) validation and (**b**) test set.

The key PI parameter is the number of permutations, i.e., the number of repetitions of random mixing of a given feature to stably estimate its impact on the ML model. PI sensitivity analysis was performed for a number of permutations between 10 and 400. PI was determined independently for validation data (Figure 13a) and test data (Figure 13b). The predictors in descending order of permutation importance are friction conditions, drawbead height, sample width, Sa of countersamples, and sample orientation (Figure 13). For the analyzed set of friction test results, the number of permutations does not significantly affect the PI value. The largest differences in PI values depending on the number of permutations occur for friction conditions. However, these differences do not exceed 3.69% and 7.37% for validation data (Figure 13a) and test data (Figure 13b), respectively. The weakest effect of sample orientation on CoF is consistent with the results presented in Figure 11c.

F-test metrics (feature ranking) assess how much a CSVM model would deteriorate if a specific explanatory variable was removed. Feature ranking allows for the analysis of which model features are most and least important when making a forecast. The F-test checks whether responses corresponding to different levels of a predictor come from populations with equal means. Importance scores correspond to:

(7) Score θ=−log(p−value)

where *p* is a measure of statistical significance.

**Figure 14:**
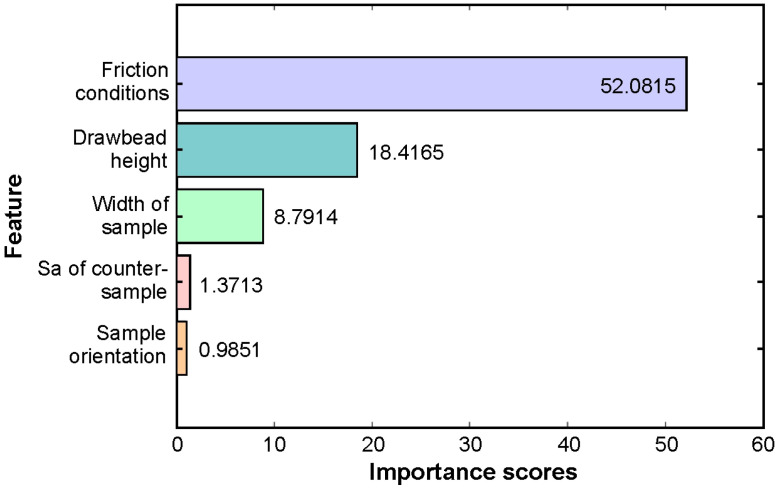
F-test importance scores for CSVM model.

A small score θ-value indicates that the corresponding predictor is not important. However, a small *p*-value indicates that a predictor is important. According to the F-test metric (Figure 14), the most significant input parameters in the CSVM model are friction conditions (*θ* = 52.0815) and drawbead height (*θ* = 18.4165).

### 3.4. SHAP Values

Shapley Additive Explanations (SHAP) is a game theory metric used to interpret ML models by quantifying the contribution of each variable to a single prediction. In game theory, Shapley Additive Explanations is a method for assigning profits among players cooperating in a coalition based on their contribution to the overall game. The Shapley value indicates how much a player should expect to gain from the overall game, given their average contribution to the game within the coalition. The SHAP value was determined from the formula [65]:

(8) $$SHAP\;value=\sum_{S\subseteq N\backslash i}\frac{\left|S\right|!\left(\left|N\right|-\left|S\right|-1\right)!}{\left|N\right|!}\left[f\left(S\cup i\right)-f\left(S\right)\right]$$

where *N* is the set of all features, *S* is a subset of features, *f*(*S* ∪ *i*) is the model prediction for feature *i* and *f*(*S*) is the model prediction when only features from subset *S* are present.

**Figure 15:**
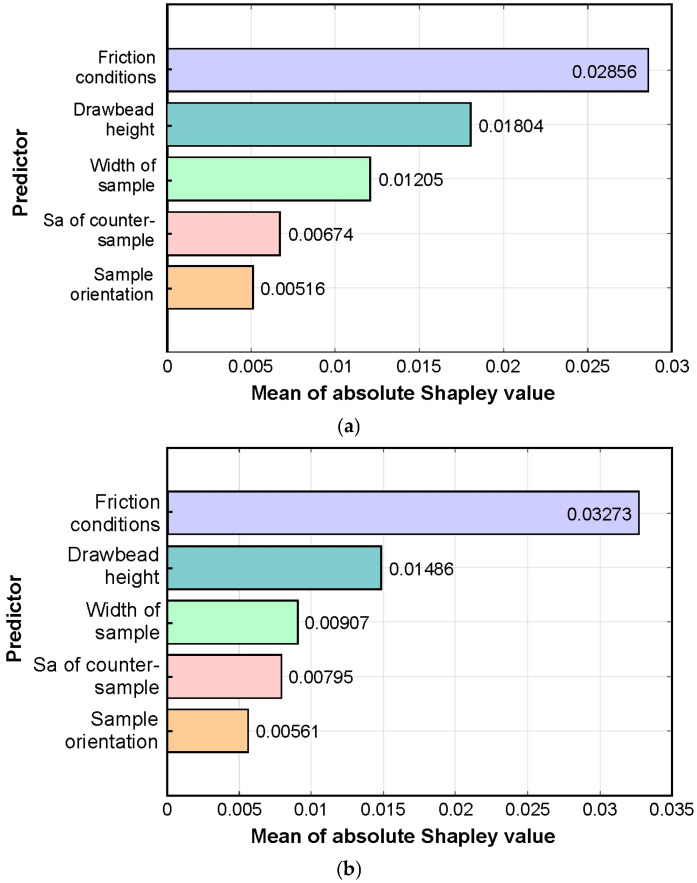
Shapley values concerning CSVM model for (**a**) training and (**b**) test set.

Figure 15 shows the influence of predictors on the mean of absolute Shapley values for the training and test data. The significance of Shapley values in both analyzed sets is consistent. This is confirmed by the statistical significance of randomly selecting data from the test set, which was not included in the training process. The categorical variable ‘friction conditions’ showed the highest significance in the training (0.02856) and test (0.03273) data sets. In order of decreasing significance, drawbead height, sample width, Sa of countersamples, and sample orientation can be listed. The order of significance of variables is consistent with the results of permutation importance (Figure 13) and F-test importance scores (Figure 14). In the case of data from the training set, the influence of drawbead height and Sa of countersamples was more dominant compared to the test data (Figure 15b).

**Figure 16:**
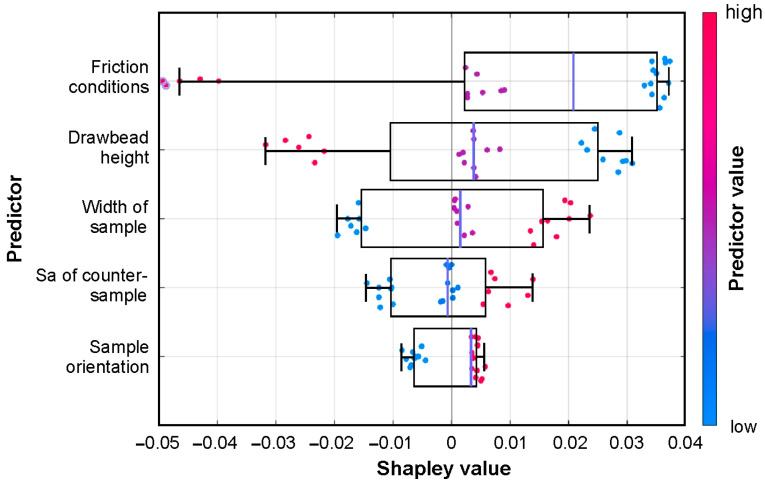
Combined swarm-box chart presenting Shapley values for CSVM model.

A combined swarm-box chart for the CSVM model (Figure 16) shows the SHAP value for each observation. The color map shows whether the variable takes high (red) or low (blue) values for a given observation. The horizontal position of points relative to the zero value indicates whether the effect of this value is associated with a higher (positive) or lower (negative) prediction. Clusters of points indicate the aggregate effect of a given variable on the CoF. Low values of the categorical variables ‘friction conditions’ (dry friction) and ‘drawbead height’ have a strong positive (increasing) effect on the CoF. Low values of the other explanatory parameters (sample width, Sa of countersamples, and sample orientation) have a negative (decreasing) effect on the CoF, but this effect is weaker compared to friction conditions and drawbead height. High values of drawbead height reduce the CoF, which is consistent with the experimental results presented in Figure 11d. Correspondingly, high values of the ‘friction conditions’ categorical parameter, i.e., lubrication with HD 1150 oil, significantly reduce the CoF value. High values of Sa of countersamples and sample width are responsible for increasing the coefficient of friction. Points corresponding to sample orientation are closest to the zero value, indicating a negligible effect of this parameter on CoF. A small value of the categorical predictor ‘sample orientation’ (samples cut according to the RD) has a decreasing effect on CoF. The opposite effect is exerted by points corresponding to a high value of the ‘sample orientation’ predictor, which is also documented in Figure 11c.

The box width corresponds to the Shapley value for a given predictor across all observations. For all explanatory variables except Sa of countersample, on average, these predictors increase the CoF. In the case of Sa of countersample, this variable only slightly reduces the CoF on average.

## 4. Conclusions

The aim of this article was to analyze the results of the drawbead simulator test using various machine learning methods to select the most appropriate algorithm and to analyze in detail the feature ranking, permutation importance, and cumulative Shapley additive explanation values of the predictors. Based on experimental and analytical investigations, the following conclusions can be drawn:

- Among the eleven ML algorithms tested, the SVM model with cubic kernel function had the lowest RMSE value (0.0085) and the highest coefficient of determination R2 (0.9657).
- The highest efficiency in CoF reduction was observed for HD 1150 oil, whose kinematic viscosity was higher than that of LAN 46 machine oil.
- Higher drawbead heights lead to a decrease in the CoF value. A higher drawbead height indicates more intense plastic deformation of the sheet material and a change in its mechanical properties due to work hardening phenomenon.
- An increase in the average surface roughness (Sa) of the countersamples leads to a trend toward an increase in the coefficient of friction.
- Based on the results of permutation importance analysis, Shapley values, and F-test importance, in descending order of influence of a given predictor on CoF, the following parameters can be listed: friction conditions, drawbead height, sample width, Sa of countersamples, and sample orientation.
- The number of permutations does not significantly affect the value of permutation importance. The largest differences in PI-values depending on the number of permutations (10–400) occur for the categorical predictor ‘friction conditions’; however, these differences do not exceed 7.37% for the test data.
- The combined swarm-box chart presenting Shapley values for the CSVM model indicates that a low value of the ‘drawbead height’ predictor has a strong, increasing effect on the CoF. However, low values of the remaining explanatory parameters (sample width, Sa of countersamples, and sample orientation) have a decreasing effect on the CoF. High Sa of countersamples and sample width contribute to an increased CoF.

The developed ML models were trained on a limited parameter space, so their ability to generalize to other materials or friction conditions requires further validation. Expanding the dataset to include other low-carbon steel sheets and surface roughness range would capture a broader spectrum of friction mechanisms and improve predictive reliability of ML model. Future research will focus on expanding the training data by conducting friction tests on other steel sheets belonging to the low-carbon DCxx steel sheet group. Considering the initial surface roughness of the sheets and their mechanical properties will allow for the development of a data-driven knowledge base supporting the design of sheet metal forming processes requiring the use of drawbeads. A further issue will be a detailed analysis of the effect of lubrication conditions, particularly the physical properties of lubricants, on friction mechanisms. Future work will also include comparing the ML models’ predictions for new input data within the range of parameters used in the training process. This will allow for a comprehensive assessment of the models’ predictive performance and generalization ability, providing reliable verification of their suitability for CoF prediction under realistic forming conditions.

## Figures and Tables

**Table 1:** Explanation of levels of categorical parameters.

Categorical Predictor	Description
Friction conditions	A—dry friction, B—lubrication with LAN-46 machine oil, C—lubrication with HD 1150 metal forming lubricant
Sample orientation relative to RD	D—sample cut along the RD, E—sample cut transversely to the RD

**Table 2:** Designations and model hyperparameters of SVM algorithms.

Kernel Function	Kernel Scale	Standardize Data	Designation
linear	automatic	yes	LSVM
quadratic	automatic	yes	QSVM
cubic	automatic	yes	CSVM
fine Gaussian	0.56	yes	FGSVM
medium Gaussian	2.2	yes	MGSVM
coarse Gaussian	8.9	yes	CGSVM

**Table 3:** Designations and model hyperparameters of regression trees.

Preset	Maximum Leaf Size	Designation
Fine tree	4	FT
Medium tree	12	MT
Coarse tree	36	CT

**Table 4:** Designations and model hyperparameters of ensemble trees.

Preset	Maximum Leaf Size	Number of Learners	Designation
Boosted tree	8	30	BOT
Bagged tree	8	30	BAT

**Table 5:** Regression statistics of analyzed ML models.

ML Model	Validation Set					Test Set					Prediction Speed (obs/s)	Training Time, s	Selected Features
	RMSE	MSE	*R* ^2^	MAE	MAPE, %	RMSE	MSE	*R* ^2^	MAE	MAPE, %			
LSVM	0.0114	0.0001	0.9326	0.0094	5.6436	0.0100	0.0001	0.9534	0.0063	4.3932	2479.10	2.1225	5/5
QSVM	0.0109	0.0001	0.9387	0.0083	4.9317	0.0100	0.0001	0.9527	0.0084	5.7707	3939.30	3.4262	5/5
CSVM	0.0126	0.0001	0.9183	0.0093	5.6752	0.0085	7.3765	0.9657	0.0070	5.0652	3706.98	2.5412	5/5
FGSVM	0.0369	0.0013	0.2984	0.0278	18.4087	0.0332	0.0011	0.4865	0.0247	18.4822	3791.36	1.7392	5/5
MGSVM	0.0130	0.0001	0.9132	0.0097	5.7789	0.0101	0.0001	0.9517	0.0077	5.6915	4217.44	1.7388	5/5
CGSVM	0.0166	0.0002	0.8582	0.0132	8.4830	0.0158	0.0002	0.8827	0.0123	9.5473	1958.78	1.6906	5/5
FT	0.0164	0.0002	0.8607	0.0123	7.1017	0.0169	0.0002	0.8663	0.0129	9.2583	4561.41	8.0392	5/5
MT	0.0226	0.0005	0.7365	0.0188	11.7682	0.0177	0.0003	0.8530	0.0143	10.1908	4950.45	5.3902	5/5
CT	0.0304	0.0009	0.5248	0.0242	15.4594	0.0275	0.0007	0.6480	0.0224	16.4663	5223.49	4.9548	5/5
BOT	0.0138	0.0001	0.9019	0.0101	5.6519	0.0120	0.0001	0.9322	0.0098	6.3821	1038.36	7.9957	5/5
BAT	0.0171	0.0002	0.8484	0.0137	8.7553	0.0147	0.0002	0.8987	0.0123	9.0283	1174.91	7.6791	5/5

## Data Availability

The original contributions presented in this study are included in the article. Further inquiries can be directed to the corresponding author.
